# Investigation of Respiratory Metastrongyloids and Other Endoparasites in Domestic Cats Living in the States of Rio de Janeiro and Rio Grande do Sul, Brazil

**DOI:** 10.3390/ani16020335

**Published:** 2026-01-22

**Authors:** Luciano Antunes Barros, Simone Morelli, Angela Di Cesare, Ananda Senhoretto do Nascimento, Sandra Márcia Tietz Marques, Lebana Fernandes Knopp, Caio dos Santos Gomes, Eduarda Nóbrega Fialho Tavares, Júlia Pereira da Silva, Aline Silva de Mattos Queiroz, Claudio Alessandro Massamitsu Sakamoto, Shihane Mohamad Costa Mendes, Tatiana Moniz Portella Lovatto, Frederic Beugnet, Karin Botteon, Donatella Damiani, Ilaria Lallone, Donato Traversa

**Affiliations:** 1 Laboratório de Apoio Diagnóstico em Doenças Parasitárias, Faculdade de Veterinaria, Universidade Federal Fluminense (UFF), Niterói 24220-900, Brazil; lucianobarrosrj@gmail.com (L.A.B.); caiogomes44@gmail.com (C.d.S.G.); claudiosakamoto@id.uff.br (C.A.M.S.); shihanem@id.uff.br (S.M.C.M.); tatianamoniz@id.uff.br (T.M.P.L.); 2 Department of Veterinary Medicine, University of Teramo, 64100 Teramo, Italy; adicesare@unite.it (A.D.C.); ddamiani1@unite.it (D.D.); ilallone@unite.it (I.L.); dtraversa@unite.it (D.T.); 3 Graduate Program of Pathology and Clinical Sciences, Universidade Federal Rural do Rio de Janeiro (UFRRJ), Seropédica 23890-000, Brazil; anandasenhoretto@gmail.com; 4 Laboratório de Helmintos, Faculdade de Veterinaria, Universidade Federal do Rio Grande do Sul (UFRGS), Porto Alegre 91540-000, Brazil; santietz@gmail.com; 5 Laboratorio de Epidemiologia Molecular, Faculdade de Veterinaria, Universidade Federal Fluminense, Niterói 24220-900, Brazil; lebanafernandes@id.uff.br; 6 Hospital Universitário de Medicina Veterinária Professor Firmino Mársico Filho, Universidade Federal Fluminense (UFF), Niterói 24220-900, Brazil; eduardafialho@gmail.com (E.N.F.T.); juliapereirasilva@id.uff.br (J.P.d.S.); mattos.aline@gmail.com (A.S.d.M.Q.); 7 Boehringer Ingelheim Animal Health, 69007 Lyon, France; frederic.beugnet@boehringer-ingelheim.com; 8 Boehringer Ingelheim Saúde Animal, São Paulo 04794-000, Brazil; karin.botteon@boehringer-ingelheim.com

**Keywords:** cat, *Aelurostrongylus abstrusus*, *Troglostrongylus brevior*, Brazil

## Abstract

The metastrongyloids *Aelurostrongylus abstrusus* and *Troglostrongylus brevior*, and the capillariid *Capillaria aerophila*, are major respiratory nematodes infecting cats. In recent decades these nematodes have been recorded in several areas of the world and have called for epizootiological studies to update knowledge on their distribution. While *A. abstrusus* is known to occur in some areas of Brazil, the presence of *T. brevior* remains uncertain, and feline infections with *C. aerophila* have never been reported. This study has investigated the occurrence of lungworms with or without other endoparasitoses in domestic cat populations of selected areas of Brazil. Individual faecal samples were collected from 537 cats, i.e., 521 in metropolitan area of Rio de Janeiro and 16 in Porto Alegre (Rio Grande do Sul). All samples were examined with Sheather’s flotation, and 521 of them also with the Baermann test. DNA was extracted from 425 Baermann sediments and molecularly examined for *A. abstrusus* and *T. brevior*. The most frequent parasites found at the copromicroscopy were Ancylostomatidae (n. 30, 5.8%), *Toxocara cati* (14, 2.7%), and *Cystoisospora felis* (n. 8; 1.5%). Larvae of *A. abstrusus* were identified by Baermann in 3 samples from Rio de Janeiro, while at PCRs, *A. abstrusus* DNA was amplified in 10 samples from Rio de Janeiro (n. 3), Seropédica (n. 3), Niterói (n. 3), and Porto Alegre (n. 1). The results indicate that *A. abstrusus* is enzootic in the investigated areas of Brazil and underline the usefulness of PCR when larvae are not detected by copromicroscopy or when only a single stool sample is analysed. Epizootiological implications, along with the absence of *T. brevior* in the examined cats and the presence of zoonotic endoparasites, are discussed.

## Simple Summary

*Aelurostrongylus abstrusus* and *Troglostrongylus brevior* are major respiratory nematodes of cats. Adults of *Aelurostrongylus abstrusus* inhabit alveoli, alveolar ducts, and bronchioles, while adults of *T. brevior* live in the bronchi and bronchioles of cats. In Brazil, extensive and updated epizootiological data on the occurrence of aelurostrongylosis and troglostrongylosis in cats are still lacking. Furthermore *T. brevior* has never been reported in cats in South America. The present study evaluated and confirmed that cats living in two states in Brazil (Rio de Janeiro and Rio Grande do Sul) are at risk of simultaneous infection with *A. abstrusus* and intestinal parasites, while *T. brevior* was not detected. The results underline the usefulness of molecular biology, especially when parasites are not detected during faecal examination or when only a single stool sample is analysed.

## 1. Introduction

*Aelurostrongylus abstrusus* and *Troglostrongylus brevior* are major respiratory metastrongyloids infecting cats in several areas of the world [1].

The lifecycle of *A. abstrusus* is indirect. Adults inhabit the alveoli, alveolar ducts, and bronchioles of felids. After mating, females lay eggs that hatch and release first-stage larvae (L1s), which reach the pharynx and are then swallowed and shed with faeces in the environment. L1s develop into third-stage larvae (L3) inside terrestrial gastropods acting as intermediate hosts, i.e., snails and slugs, and cats become infected by ingesting either intermediate hosts or, more frequently, paratenic hosts, e.g., small birds, rodents, and reptiles [1].

Adult *T. brevior* live in the bronchi and bronchioles of cats [1] and their lifecycle overlaps that of *A. abstrusus*, though it is shorter. Additionally, a vertical transmission route has been described, most likely via milk [2].

*Capillaria aerophila* is another main respiratory parasite of cats (and dogs) worldwide [3]. It has a direct lifecycle, with adult parasites that are localised in the mucosa of the trachea and large bronchi [1]. After mating, females lay eggs, which are carried to the pharynx via coughing and are swallowed and released in the environment with faeces. Thereafter, eggs mature, and cats become infected after ingesting the larvated eggs, or earthworms, which can serve as facultative intermediate or paratenic hosts [1].

Lungworm infections in cats may be subclinical or present with mild to severe respiratory signs, e.g., cough, dyspnea, tachypnea, and/or nonspecific signs, e.g., lethargy, weight loss, and anorexia [1]. Importantly, troglostrongylosis may cause severe and potentially life-threatening clinical signs, especially in kittens and young cats [1].

After the first detection of *A. abstrusus* in domestic cats in Rio Grande do Sul [4], in the last decade, the nematode has been reported in different areas of Brazil, e.g., Amazonia, Vilhena, Porto Alegre, and Barra do Piraí [5,6,7,8]. Also, in Brazil, *A. abstrusus* has been found in wild-caught snails, e.g., *Lissachatina fulica*, *Latipes erinaceus*, and *Diplosolenodes occidentalis* [9,10,11,12].

The European wildcat is the natural reservoir of *T. brevior*, though it has been recently recognised as a cause of respiratory disease in domestic cats [13,14]. Feline *T. brevior* infections have never been reported in Brazil, though larval stages have been detected in *L. fulica* in Colombia [15]. *Capillaria aerophila* has never been detected in Brazil, although it is present in South America [16].

Multiple factors may be involved in the emergence of respiratory nematodes in different geographic areas, including South America, e.g., movements of pets and intermediate and/or paratenic hosts, urbanisation, rising temperatures, and a reduction in the natural habitats of wildlife [3,13,17]. However, lungworms are still overlooked in many settings for a range of reasons, e.g., limited clinical awareness, absence of routine surveillance programmes, and limited application of an appropriate diagnostic approach, as also pointed out in some studies carried out in Brazil [6,8]. Copromicroscopic techniques are currently considered the gold standard for the diagnosis of lungworm infections in cats., specifically Baermann’s test, which allows the detection of L1s of *A. abstrusus* and *T. brevior*, while faecal flotation is used for the identification of *C. aerophila* eggs [1].

Many studies have shown that lungworms are often detected in cats that are infected with other endoparasites. Diverse categories of parasites occur simultaneously in the same cat populations of Brazil, which are thus at risk of being co-infected with respiratory and intestinal parasites [18,19,20]. This epizootiological feature is due to the fact that many small animals that are preyed on by cats are intermediate or paratenic hosts of other helminths or protozoa. Mixed infections are thus frequent in cats, especially when they prey on small animals. Therefore, routine and continuous epizootiological monitoring with appropriate copromicroscopic methods is warranted in cats that could be infected with both respiratory and intestinal parasites to appropriately use broad-spectrum parasiticides. Accordingly, the present study has investigated the occurrence of lungworms, along with the simultaneous presence of other major felid parasites, in cats living in Rio de Janeiro and Rio Grande do Sul through copromicroscopic and molecular tests.

## 2. Materials and Methods

**Figure 1:**
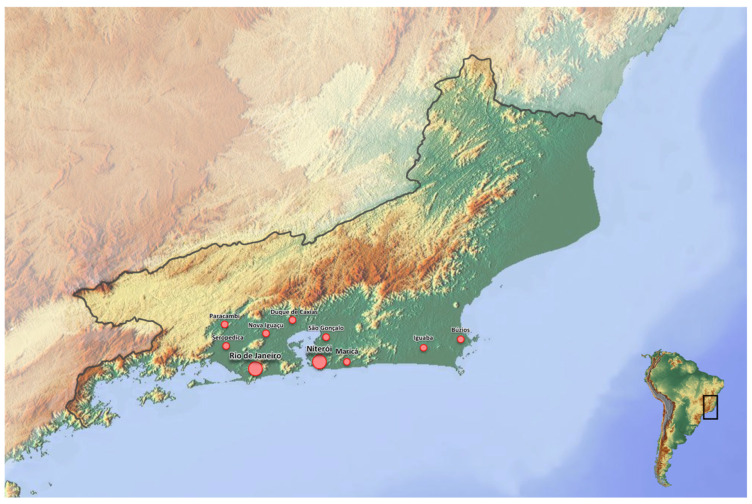
Geographic map indicating the geographical areas sampled in the State of Rio de Janeiro. The size of the red circles is related to the number of cats included, i.e., the bigger the circle, the higher the number of cats enrolled in the study.

A total of 537 individual faecal samples were collected from privately owned cats living in Brazil in the State of Rio de Janeiro (i.e., metropolitan area of Rio de Janeiro, n. 521, Figure 1) and Rio Grande do Sul (i.e., Porto Alegre, n. 16). Of the 537 cats examined, 320 (59.6%) were females and 217 (40.4%) were males, and their age ranged from 2 months to 20 years. The median age of the cats included in the study was 4 years. Overall, 288 (53.6%) cats were <4 years old, while 249 (46.4%) were ≥4 years old. Of them, 305 (56.8%) cats had an indoor lifestyle with occasional access to the outdoors, 172 (32%) lived only indoors without outdoor access, and 60 (11.2%) lived permanently outdoors.

All samples were microscopically examined using Baermann’s technique (n. 537) to detect metastrongyloid L1 and Sheather’s flotation for the detection of other endoparasites.

Four hundred and twenty-four (n. 424) Baermann sediments, i.e., n. 408 from Rio de Janeiro and n. 16 from Rio Grande do Sul, were subjected to two separate nested PCRs specific to *A. abstrusus* and *T. brevior*, as previously described. Specifically, genomic DNA was extracted from each sample with a commercial kit (i.e., Exgene Stool SV, GeneAll Biotechnology Co., Ltd., Seoul, Republic of Korea) and amplified in a nested PCR using a set of primers universal for strongylid nematodes (i.e., NC1–NC2) for the first step, while in the second step, a diagnostic primer set, AabFor-AabRev or TbrFor-TbrRev, was used to amplify a specific internal region, within the rDNA ITS2, of ∼233 bp and ∼356 bp of *A. abstrusus* and *T. brevior*, respectively [21,22].

Obtained amplicons were sequenced and aligned using Data Analysis in Molecular Biology and Evolution version 4.5.55 (DAMBE). The sequences obtained were aligned and then compared with those available in the GenBank TM using the Nucleotide–Nucleotide “Basic Local Alignment Search Tool” (BLAST, version 2.17.0).

## 3. Results

### 3.1. Baermann’s Method

In total, six cats scored positive for *A. abstrusus* L1, three from Rio de Janeiro, one from Niterói, and two from Seropédica, [Table 1]. One cat from Porto Alegre scored positive for nematode larvae at the Baermann’s method, though they were not identified due to sample deterioration, probably related to transport and/or storage conditions.

### 3.2. Sheather’s Flotation

Using Sheather’s flotation, the most frequently detected parasites were Ancylostomatidae, *Toxocara cati*, and *Cystoisospora felis*, found in 30 (5.6%), 14 (2.6%), and 8 (1.5%) faecal samples, respectively.

The three cats positive for *A. abstrusus* larvae had monospecific infections, while nine cats (1.7%) were co-infected with more than one intestinal parasite, *T. cati* + Ancylostomatidae being the most frequent combination (n. five cats). The detailed copromicroscopy results are shown in Table 2 and Table 3.

### 3.3. Molecular Analyses

Ten (2.4%) cats were genetically positive for *A. abstrusus*. This included the six cats who scored positive using the Baermann technique (Seropédica (n. one), Niterói (n. two) and Rio de Janeiro (n. three)), and the cat from which unidentified L1 were recovered (from Porto Alegre).

Five cats positive for *A. abstrusus* DNA (1.2%) were also copromicroscopically positive for other parasites, i.e., three cats from Rio de Janeiro were co-infected with *T. cati* (Rio de Janeiro), and two were co-infected with Ancylostomatidae (Niterói) [Table 3].

Sequences were generated from all 10 A. abstrusus amplicons. Of them, six sequences, i.e., three from Rio de Janeiro, two from Niterói, and one from Seropédica, had 100% homology with an isolate of A. abstrusus detected from Colombia (MH779453). The four remaining isolates, i.e., two from Seropédica, one from Porto Alegre, and one from Niterói, showed 100% homology with an A. abstrusus isolate from the Brazilian Amazon (MZ093629).

## 4. Discussion

These data indicate that *A. abstrusus* occurs in cat populations in two areas of Brazil, i.e., the states of Rio de Janeiro and Rio Grande do Sul, despite a recent epizootiological investigation carried out in Rio de Janeiro in which no cats infected with *A. abstrusus* were detected [23].

A comparison between the present and past results obtained in Brazil is difficult. Data on the occurrence of *A. abstrusus* in the Rio de Janeiro area are scant and fragmentary, as in recent decades, this lungworm has been described in case reports or single isolated studies [7,24,25,26]. The low number of cats included herein from the Porto Alegre region prevents further comparisons with other studies. Regardless, the results confirm the stable occurrence of this lungworm in this region of Brazil, as shown in a study carried out more than 10 years ago [5].

The current scenario suggests a general lower prevalence of feline aelurostrongylosis in the study areas examined in this study and Brazil in general when compared to Europe [27,28,29,30]. This difference may depend on different factors, i.e., the overall number of cats included in previous studies, the collection of multiple faecal samples, and the inclusion of a higher proportion of cats living predominantly outdoors compared to the present study [5,6,7].

Extensive epizootiological data on the occurrence of *A. abstrusus* in cats in Brazil are still lacking, though it can be hypothesised that the demo-geographics of Brazil, e.g., the lower density of the cat population in Brazil, and the subsequent high dispersion of domestic cats in its wide territories compared to Europe [31,32], may limit interactions between intermediate hosts and contaminated environments, thereby reducing the risk of lungworm transmission. Under an epizootiological standpoint, the housing and lifestyle of the herein-studied cats could also explain the high proportion of lungworm-negative animals. In fact, around half of the sampled cats lived indoors with occasional access to a garden or a yard, thus having reduced contact with intermediate or paratenic hosts in a densely urban setting. In this study, only two cats positive for *A. abstrusus* had a predominantly outdoor lifestyle, while five were indoor-housed cats with limited access to the outdoors, and three were permanently indoors. Therefore, these findings confirm that cats may be exposed to the risk of infection even when living in apartments [33,34], though they are less exposed to intermediate or paratenic hosts compared to cats with limited access to the outdoors or permanently outdoor cats [1,3]. The fact that infection with *A. abstrusus* was predominantly observed in cats of <4 years (80% of positive cats) is likely influenced by a combination of factors, including less developed immunity compared to the older cats, and behavioural differences such as increased exploration and a higher tendency to engage in predatory activities [35,36].

Diagnostic methods are another factor that may impact the results of epizootiological studies on lungworms. False negative results from faecal examinations may occur due to a low number of L1 in faeces, intermittent larval shedding, examination of only a single faecal sample, or the presence of unidentifiable larvae [1]. This inherent limitation of conventional copromicroscopy is confirmed by the present study’s results. In fact, seven samples that were negative based on Baermann’s test were positive for *A. abstrusus* when examined molecularly. Accordingly, recent studies have already shown that molecular tests conducted on faecal or pharyngeal samples may reveal positive samples that score negative upon faecal examination [1].

The present data confirm that different *A. abstrusus* isolates and (possibly) haplotypes circulate among cat populations in Brazil and in South America in general [6,8,15,37]. At present, implications related to the existence of isolates presenting SNPs and to different haplotypes are not known. It cannot be excluded that different haplotypes may have different pathogenic features. A fatal case of hemorrhagic meningoencephalitis due to ectopic localization of *A. abstrusus* has been described in Barra do Piraí, Rio de Janeiro, Brazil [8]. There is merit in further investigating the molecular features of this lungworm and the eventual occurrence of strains/haplotypes with pathogenic peculiarities, e.g., capability/tendency to migrate in the central nervous system.

The lack of *T. brevior* larvae or DNA in the faeces of the studied cats could be due to a range of reasons. A true absence of the parasite in the examined areas is reasonable, as this nematode has never been found in previous similar surveys in Brazil [7,18,38]. Also, the absence of the European wildcat, which is considered the natural host of *T. brevior* [13], in South America, including in Brazil, may suggest a true absence of this lungworm.

Nevertheless, *T. brevior* L1 were found in snails from Colombia [15], thus suggesting that it could be present in some regions where species of wild felids may act as natural reservoirs, such as the European wildcat in Europe [13]. As another hypothesis, the recent introduction of the parasite in South America (e.g., via the movements of cats and intermediate and/or paratenic hosts) could also be possible, thus indicating potential spreading of troglostrongylosis in domestic cats in South America, including Brazil, as already discussed elsewhere [13]. On the whole, the occurrence of *T. brevior* in feline populations living in South and North America has not been demonstrated yet. On the other hand, past studies have shown infections by a closely related crenosomatidae, i.e., *Troglostrongylus wilsoni*, in bobcats (*Lynx rufus*) and in Canadian lynxes (*Lynx canadensis*) living in North America [39,40,41]. Although only rarely reported in these two wild felid species, it cannot be excluded that this nematode may undergo a spill-over and spread into domestic cat populations, similarly to *T. brevior* in Europe [13].

Among respiratory parasites, *C. aerophila* was not found in cats in the present study. To this day, no cases of feline or canine capillariosis have been documented in Brazil. However, infections have been reported in both wild and domestic felids and wild canids in other countries in South America, i.e., Chile, Bolivia, and Uruguay [16,42,43]. Therefore, a future increase in clinical cases is possible, and continuous monitoring is necessary.

With regard to the other endoparasites detected, it is worthy of note that the potentially zoonotic *Ancylostoma* spp. and *T. cati* were the two most frequently diagnosed nematodes in privately owned cats included in this study, in addition to *Strongyloides* sp. The occurrence of these parasites is not surprising, as they have already been recorded in previous studies conducted in Brazil [18,44,45], and in different studies, hookworms and roundworms were among the most frequently detected [18,44,45]. Similarly, infections with *C. felis. Trichuris* spp. and *Dipylidiym caninum* were also expected, and the presence of these parasites has been largely described in previous studies [23,45,46,47]. The finding of adult *L. radovskyi* was accidental, as sometimes parasitic stages of mites may be found in the faeces of cats when ingested during cat self-grooming.

The same and/or similar parasitic species have also been documented to occur in wild felids, e.g., *Leopardus colocolo*, *Leopardus guttulus*, and *Puma concolor*, in Brazil. Infections with *A. abstrusus*, *Ancylostoma* spp., *Toxocara* spp., and *C. felis* have been documented, suggesting that wildlife species probably contribute to (i) the maintenance of the lifecycle of these parasites in peri-urban ecosystems, and (ii) enhancing the possibilities of transmission to other wild and domestic animals, and to humans in the case of zoonotic parasites [48,49].

It is worth noting that the majority of cats in this study that were positive for parasites were housed indoors and had limited access to the outdoors. Relatively high percentages of endoparasite positivity in indoor cats were also detected in other past studies carried out in different countries, including Brazil [23,50]. It has been evidenced that cat owners may lack attention towards indoor cats in terms of antiparasitic preventative measures, as many of them believe that such cats are not/very uncommonly exposed to the risk of becoming infected with parasites [51].

Therefore, a higher standard of owner education by veterinarians and the implementation of adequate control plans for both outdoor and indoor cats are advocated for.

Nevertheless, the potential health implications for domestic cats and the possible zoonotic risks associated with these parasites and others detected in this study (e.g., *D. caninum* and *T. gondii*) clearly indicate that improved epizootiological surveillance and control measures are warranted. Future studies should aim to include larger, more geographically representative sample sizes and integrate molecular diagnostic tools to improve sensitivity. Such efforts are essential to better understand the epizootiology of these parasites and to develop effective prevention and control measures.

The present data confirm that cats living in the examined areas are at simultaneous risk of infection by *A. abstrusus* and intestinal parasites, with some of the latter being potentially zoonotic. Though not extensively reported in Brazil, data from Europe demonstrated that co-infections with lungworms and intestinal parasites are frequent [19,20] due to shared transmission patterns. For example, the presence of cats co-infected with *A. abstrusus* and *T. cati* suggests that cats may prey on paratenic hosts, e.g., rodents, that may harbour and transmit both species at the same time [52]. Overall, the present data confirm that cats may favour the introduction, circulation, and spread of different categories of parasites with zoonotic potential in a domestic environment.

## 5. Conclusions

In conclusion, this study confirms the presence of *A. abstrusus* in cats in Brazil and highlights the key role of PCR in epizootiological surveys, particularly when only a single faecal sample is available for compromicroscopy. Further studies are also advocated for to understand the true epizootiological context of *T. brevior* in South America, i.e., if it is present and undetected, or if it has yet to be truly established in these territories. Hence, large-scale epizootiological studies involving wildlife are essential to determine the true prevalence of feline lungworm in Brazil and to implement specific control strategies. This is particularly true considering that cats may be co-infected with lungworms and other nematodes/cestodes simultaneously, with subsequent complication of the clinical picture resulting in impaired diagnosis and difficulties in the diagnostic process and case management. This calls for the implementation and proper use of broad-spectrum parasiticides available on the market for the control of cat helminthic infections.

## Figures and Tables

**Table 1:** Number (n) and percentage (%) of positive samples based on Baermann’s technique in the different municipalities of Rio De Janeiro and in Porto Alegre (Rio Grande do Sul) examined in this study.

Municipalities	n. Cats	*Aelurostrongylus abstrusus* n. pos (%)	*Strongyloides* spp. n. pos (%)	Unidentifiable n. pos (%)
Búzios	1	-	-	-
Duque de Caxias	26	-	-	-
Iguaba	3	-	-	-
Maricá	9	-	-	-
Niterói	259	1 (0.4)	1 (0.4)	-
Nova Iguaçu	4	-	-	-
Paracambi	1	-	-	-
Porto Alegre	16	-	-	1 (6.25)
Rio de Janeiro	202	-	-	-
São Gonçalo	5	-	-	-
Seropédica	11	2 (18.2)	-	-
Tot	537	3 (0.6)	1 (0.2)	1 (0.2)

**Table 2:** Number (n) and percentage (%) of positive samples based on Sheater’s flotation in the different municipalities of Rio De Janeiro and in Porto Alegre (Rio Grande do Sul) examined in this study.

Municipalities	n. Cats	Ancylostomatidae n. pos (%)	*Toxocara cati* n. pos (%)	*Trichuris* spp. n. pos (%)	*Dipylidium caninum* n. pos (%)	*Platynosomum fastosum* n. pos (%)	*Cystoisospora felis* n. pos (%)	*Lynxacarus radovskyi* n. pos (%)
Búzios	1	-	-	-	-	-	-	-
Duque de Caxias	26	6 (23)	-	-	-	-	-	-
Iguaba	3	-	-	-	-	-	-	-
Maricá	9	-	-	-	-	-	-	-
Niterói	259	22 (8.5)	11 (4.2)	-	3 (1.2)	4 (1.5)	8 (3.1)	1 (0.4)
Nova Iguaçu	4	-	-	-	-	-	-	-
Paracambi	1	-	-	-	-	-	-	-
Porto Alegre	16	-	-	-	-	-	-	-
Rio de Janeiro	202	2 (1)	3 (1.5)	-	-	-	-	-
São Gonçalo	5	-	-	1 (20)	-	-	-	-
Seropédica	11	-	-	-	-	-	-	-
Tot	537	30 (5.6)	14 (2.6)	1 (0.2)	3 (0.6)	4 (0.7)	8 (1.5)	1 (0.2)

**Table 3:** Parasitic co-infections detected in cats in the present study.

Co-Infection	n. Cats
*Aelurostrongylus abstrusus* + *Toxocara cati*	3 *
*Aelurostrongylus abstrusus* + Ancylostomatidae	2 *
*Toxocara cati* + Ancylostomatidae	5
Ancylostomatidae + *Platynosomum fastosum*	2
*Toxocara cati* + *Dipylidium caninum*	1
*Toxocara cati* + *Cystoisospora felis*	1

* *Aelurostrongylus abstrusus* infection only detected via DNA detection in PCR and not through Baermann’s examination.

## Data Availability

All the data generated in the present study have been included in the present manuscript.
